# Clinical Characteristics and CD4^+^ T Cell Subsets in IgG4-Related Disease 

**DOI:** 10.3389/fimmu.2022.825386

**Published:** 2022-04-01

**Authors:** Yan Yang, Chen Wang, Lei Shi, Shuoran Yang, Yan Liu, Jing Luo, Caihong Wang

**Affiliations:** ^1^ Department of Rheumatology and Immunology, The Second Hospital of Shanxi Medical University, Taiyuan, China; ^2^ Department of Pathology, The Second Hospital of Shanxi Medical University, Taiyuan, China; ^3^ Department of Oral and Maxillofacial Surgery, The Second Hospital of Shanxi Medical University, Taiyuan, China; ^4^ Department of Rheumatology Laboratory, The Second Hospital of Shanxi Medical University, Taiyuan, China

**Keywords:** IgG4-related disease, atopy, eosinophils, CD4^+^ T cell subsets, cytokines

## Abstract

**Objectives:**

To characterize the clinical features of IgG4-related disease (IgG4-RD) and analyze the peripheral T lymphocyte subsets and cytokine levels.

**Methods:**

A total of 52 patients with newly diagnosed IgG4-RD were enrolled in the retrospective study. Baseline clinical characteristics and examinational findings were systematically reviewed.

**Results:**

IgG4-RD patients had a male predominance, with an average age of 57.4 ± 10.3 years (range 27-81). The mean number of involved organs was 2.7 (range 1-8). Submandibular gland (57.7%) and lacrimal gland/orbit (40.4%) were the most commonly involved organs. Serum IgG4 increased in 97.9% of the patients, the median level was 1300 (585.25, 1975) mg/dl. Decreased C3 and C4 accounted for 77.8% and 55.6% of this patient cohort, respectively. Receiver operating characteristic (ROC) test indicated the possibility of lung/pleura involvement when C3 was less than 0.570 g/l (AUC = 0.788, P = 0.014), and kidney involvement when C3 was less than 0.545 g/l (AUC = 0.796, P = 0.014). Compared with healthy controls (HC), the absolute Th1 counts were higher in IgG4-RD patients (157.58 cells/μl *vs.* 130.54 cells/μl, P = 0.038), while the absolute counts of Th2, Th17 and T regulatory (Treg) cells, as well as Th17/Treg ratio were not statistically different. The levels of serum IL-4, IL-6, IL-10, IL-17, TNF-α, and IFN-γ were higher in patients with IgG4-RD as compared with HC (P < 0.001). Serum IL-10 was positively correlated with IL-4, TNF-α and IFN-γ, but uncorrelated with Treg cells. Serum IgG4 level was positively associated with the number of affected organs, eosinophil counts, and ESR, whereas inversely associated with C3, C4, IgM, and IgA.

**Conclusion:**

Submandibular and lacrimal glands are the most commonly involved organs in IgG4-RD. Serum C3 level could be a predictor of lung/pleura and kidney involvement in the disease process. Elevated Th1 cells are probably related to chronic inflammation and fibrosis. Treg cells are unlikely to play an important role in the pathogenesis of IgG4-RD.

## Introduction

IgG4-related disease (IgG4-RD) is an immune-mediated chronic fibro-inflammatory disease characterized by enlargement and sclerosis of affected organs, compression of the surrounding tissues, and ultimately leads to tissue destruction and organ failure. Dense lymphocytic infiltration, especially dramatic IgG4^+^ plasma cells, and storiform fibrosis are the significant pathological features of the disease (1–3).

It is a highly heterogeneous disease that can affect virtually all organs, and the clinical manifestations are protean. Pancreas, bile duct, lacrimal and salivary glands, orbital and retroperitoneal tissues are the commonly affected sites. In addition, IgG4-RD is an excellent imitator, which is easily confused with malignancy, infection, and other immune-mediated conditions, and likely to result in misdiagnosis or even over-diagnosis. Thus, the accurate diagnosis of IgG4-RD is highly dependent on the comprehensive analyses of clinical features, laboratory findings, imaging examinations, and histopathological manifestations.

In this study, we compared demographic characteristics and recorded the laboratory parameters of 52 IgG4-RD patients, and simultaneously analyzed peripheral lymphocyte subgroups, CD4^+^ T cell subsets, and serum cytokines. We expect these data would improve the understanding of IgG4-RD and provide a reference for exploring pathogenesis and therapeutic targets.

## Materials and Methods

### Patient Enrollment

This retrospective observational study enrolled 52 patients with IgG4-RD referred to the Second Hospital of Shanxi Medical University from November 2015 to January 2021. According to the classification criteria for IgG4-RD formulated by the American College of Rheumatology (ACR) and European League Against Rheumatism (EULAR) in 2019 (4), the newly diagnosed and untreated patients were classified following a sequentially three-step that consist of entry criterion, exclusion criteria and inclusion criteria. This study was approved by the Medical Ethics Committee of the second hospital of Shanxi Medical University (Shanxi, China).

### Patient Characteristics and Examination Parameters

Patient characteristics including age at diagnosis, gender, disease duration, and atopic history were recorded, along with clinical, radiologic, laboratory, and pathologic data obtained at the time of presentation. The HC consisted of 50 healthy volunteers with gender and age matched during the same period. Atopic history was collected according to the definitions of the European Academy of Allergy and Clinical Immunology (EAACI) (5).

Laboratory data comprised blood count, liver and renal function, erythrocyte sedimentation rate (ESR), C-reactive protein (CRP), and complement (C3, C4). Serum immunoglobulin (IgG, IgA, and IgM) and IgG subclasses (IgG1, IgG2, IgG3, and IgG4) were detected by immunoturbidimetry. Rheumatoid factor (RF) was determined by enzyme-linked immunosorbent assay (ELISA), anti-nuclear antibody (ANA) and anti-extractable nuclear antibodies (anti-ENAs) were measured by indirect immunofluorescence assay (IFA) and western blotting, respectively.

Immunophenotypes of lymphocyte subsets were determined by FACSCalibur (BD Biosciences). All immunofluorescence antibodies were purchased from BD Biosciences. The absolute counts of peripheral lymphocyte subgroups (CD3^+^ T/CD4^+^ T/CD8^+^ T/CD19^+^ B/NK cells) were analyzed by BD MultiSET software and FACSCanto II (BD Immunocytometry Systems, USA). For CD4^+^ T cell subsets (Th1/Th2/Th17/Treg cells), we needed to gate on CD4^+^ T cells initially. First, we added Ionomycin, PMA, and GolgiStop into 200μl heparin-anticoagulated venous blood to stimulate cells. Second, cells were labeled by CD4-FITC, followed by 2ml freshly prepared Fixation/Permeabilization. Finally, we stained cells using IFN-γ-APC/IL-4-PE/IL-17-PE to identify Th1/Th2/Th17 cells. Treg cells in 80μl heparin-anticoagulated venous blood were labeled by CD4-FITC and CD25-APE, followed by 1ml freshly prepared Fixation/Permeabilization, and then stained by anti-FoxP3-PE. The data were analyzed by the BD CellQuest Pro software. The calculation formula is that the number of CD4^+^ T cell subsets = the number of CD4^+^ T cells * the percentage of CD4^+^ T cell subsets.

Serum cytokines (IL-2, IL-4, IL-6, IL-10, IL-17, TNF-α, and IFN-γ) were detected by magnetic bead-based multiplex immunoassay using Human Th1/Th2/Th17 subgroup test kit (Jiangxi Cellgene Biotechnology Co., Ltd China) according to the manufacturer’s recommendations. The Bio-Plex 200 reader was used to acquire the data as Median Fluorescence Intensity (MFI) and concentration (pg/ml).

Imaging examinations included ultrasonography, computerized tomography (CT), enhanced CT, magnetic resonance imaging (MRI), and 18F-fluorodeoxyglucose positron emission tomography/computed tomography (PET/CT). Pathological and immunohistochemical examinations were carried out on 39 patients with IgG4-RD.

### Statistical Analysis

Data for patient characteristics and laboratory findings were summarized as the median and interquartile range (IQR) unless otherwise stated. T-test or rank-sum test was used to compare the measurement data between groups. The chi-square test of independence was used to assess categorical variables. Spearman’s rank correlation was used to analyze the correlation between the measurement data of the patient group. ROC curve was used to analyze the correlation between serum C3 level and the affected organs, and the predictive effect was evaluated according to area under the curve (AUC). P < 0.05 indicates a statistical difference. The statistical software is R 4.0.5.

## Results

### Patient Characteristics

A total of untreated 52 IgG4-RD patients, 30 males and 22 females (M: F 1.36:1), were enrolled in this study. The demographic characteristics are shown in **Table 1**. Mean age at diagnosis was 57.4 ± 10.3 years (range 27-81). Median time from onset to diagnosis was 12 (3, 9) months.

**Table 1:** Comparisons of clinical characteristics in patients with IgG4-RD in different studies.

Characteristics	Current study	Zhang et al. (6)	Yamada et al. (7)	Wallace et al. (8)
Number of cases (n)	52	346	334	125
Age at diagnosis, mean (range)	57.4 (27-81)	53.8 (9-83)	63.8 (25-91)	55.2 (24-83)
Disease duration (months)	12 (3, 24)	12 (3-36)	–	5.2 ± 8.5^a^
Male: Female	1.36:1	1.98:1	1.59:1	1.55:1
Race	Chinese	Chinese	Japanese	76%Caucasian
Elevated serum IgG4, n (%)	47 (97.9)	285 (94.1)	318 (95.5)	52 (51.0)
Serum IgG4 level (mg/dl)	1300 (585.25, 1975)	766 (313, 1780)	755 ± 642	–
Number of affected organs, mean (range)	2.7 (1-8)	–	3.2 (1-11)	2.3 (1-7)
History of atopy, n (%)	11 (21.2)	172 (49.7) ^b^	–	–

Data are expressed as mean ± S.D., M (P_25_, P_75_) or number (%). ^a^Years of disease duration; ^b^History of allergy.

### Organ Involvement

In our cohort, 10 patients (19.2%) and 23 patients (44.2%) had 1 and 2 organs involved, respectively, 19 patients (36.6%) had more than 2 organs involved. The mean number of affected organs was 2.7 (range 1-8) (**Table 1**). Submandibular gland (30, 57.7%), lacrimal gland/orbit (21, 40.4%), lung/pleura (16, 30.8%) were the most commonly affected organs, followed by pancreas (14, 26.9%), parotid (12, 23.1%), bile duct (11, 21.2%), kidney (11, 21.2%), nasal cavity/sinuses (8, 15.4%), retroperitoneal fibrosis (4, 7.7%), and others. Notably, vaginal involvement was detected by PET/CT which showed hypermetabolic nodules in the left lateral wall in a multi-organ involved woman (**Table 2**).

**Table 2:** Comparisons of organ involvement in patients with IgG4-RD in different studies.

Affected Organs	Current study (n=52)	Zhang et al. (6) (n=346)	Yamada et al. (7) (n=334)	Wallace et al. (8) (n=125)
Submandibular gland	30 (57.7)	182 (52.6)	242 (72.7)^a^	35 (28.0)
Parotid	12 (23.1)	75 (21.7)		21 (16.8)
Lacrimal gland/Orbit	21 (40.4)	161 (46.5)	190 (57.1)	28 (22.4)
Lung/Pleura	16 (30.8)	97 (28.0)^b^ + 21 (6.1)^c^	78 (23.4)	22 (17.6)
Pancreas	14 (26.9)	133 (38.4)	85 (25.5)	24 (19.2)
Kidney	11 (21.2)^d^	24 (6.9)	79 (23.7)	15 (12.0)
Bile duct	11 (21.2)	88 (25.4)	18 (5.4)	12 (9.6)
Nasal cavity/Sinuses	8 (15.4)	81 (23.4)	–	3 (2.4)^e^ + 5 (4.0)^f^
RPF	4 (7.7)	69 (19.9)	83 (24.9)^g^	23 (18.4)
Aorta	2 (1.9)	33 (9.5)		14 (11.2)
Skin	3 (5.8)	22 (6.4)	5 (1.5)	2 (1.6)
Prostate	2 (3.8)	48 (20.9)	32 (9.6)	4 (3.2)
Thyroid	1 (1.9)	14 (4.0)	3 (0.9)	7 (5.6)
Meninges	1 (1.9)	5 (1.4)	–	3 (2.4)
Hypophysis	1 (1.9)	8 (2.3)	–	–
Mesentery	1 (1.9)	9 (2.6)	–	2 (1.6)
Breast	1 (1.9)	–	–	–
Vagina	1 (1.9)	–	–	–

Data are expressed as number (%). ^a^The data included both submandibular gland and parotid involvement; ^b^Lung; ^c^Pleura; ^d^Kidney involvement included kidney, renal pelvis, and ureter involvement; ^e^Nasal cavity; ^f^Sinuses; ^g^The data included both RPF and Aorta involvement; RPF, Retroperitoneal fibrosis.

### Atopy

There were 11 patients (21.2%) with atopic history (**Table 1**). The main manifestations were asthma and rhinoconjunctivitis. The allergens were mostly pollen, wormwood, mites and dust. There was no significant difference in serum IgG4 level, absolute Th2 counts, and peripheral eosinophil counts between atopic and non-atopic patients (**Table 3**).

**Table 3:** Comparisons of eosinophils, Th2 and serum IgG4 level in atopic and non-atopic patients with IgG4-RD.

Group	Eosinophils (*10^9^/l)		Th2 (cells/μl)		IgG4 (mg/dl)	
	N	M (P_25_, P_75_)	N	M (P_25_, P_75_)	N	M (P_25_, P_75_)
non-Atopy	41	0.22 (0.10, 0.47)	27	7.40 (5.35, 11.48)	38	1155 (408, 2120)
Atopy	11	0.28 (0.26, 0.43)	9	8.61 (5.84, 15.09)	10	1345 (1140, 1590)
*P-*value		0.494		0.523		0.780

### Laboratory Examinations

In this study, 9 patients (9/52, 17.3%) had elevated eosinophil counts. 33 patients (33/46, 71.7%) had mild-to-moderate elevated ESR with a median of 27 (15.75, 57.75) mm/h; CRP increased in 12 patients (12/40, 30.0%) with a median of 3.14 (2.81, 8.32) mg/l. There were 47 patients (47/48, 97.9%) who had elevated serum IgG4, with a median of 1300 (585.25, 1975) mg/dl. The patients with elevated IgG1, IgG2, and IgG3 were 4 (4/27, 14.8%), 12 (12/26, 46.2%), and 5 (5/27, 18.5%), respectively. Decreased C3 occurred in 21 patients (21/27, 77.8%), and 15 patients (15/27, 55.6%) had C4 reduction. Low titers of ANA were detected in 6 cases (6/50, 12.0%), 11 cases (11/50, 22.0%) with positive RF, 1 case with positive anti-smooth muscle antibody, 1 case with positive anti-MCV antibody, and the anti-ENAs were all negative (**Table 4**).

**Table 4:** Laboratory findings in patients with IgG4-RD.

Laboratory Examination	Value
ESR (mm/h)	27 (15.75, 57.75)
Elevated ESR, n (%)	33 (71.7)
CRP (mg/l)	3.14 (2.81, 8.32)
Elevated CRP, n (%)	12 (30.0)
Elevated eosinophils, n (%)	9 (17.3)
C3 < 0.79 g/l, n (%)	21 (77.8)
C4 < 0.16 g/l, n (%)	15 (55.6)
ANA positive, n (%)	6 (12.0)
RF positive, n (%)	11 (22.0)
IgG1 (mg/dl)	892 (675.5, 1100)
Elevated IgG1, n (%)	4 (14.8)
IgG2 (mg/dl)	669.5 (450.25, 791.75)
Elevated IgG2, n (%)	12 (46.2)
IgG3 (mg/dl)	48 (32.7, 91.4)
Elevated IgG3, n (%)	5 (18.5)
IgG4 (mg/dl)	1300 (585.25, 1975)
Elevated IgG4, n (%)	47 (97.9)

Data are expressed as M (P_25_, P_75_) or number (%). ESR, Erythrocyte sedimentation rate; CRP, C-reactive protein; C3, Complement 3; C4, Complement 4; ANA, anti-nuclear antibody; RF, Rheumatoid factor; Ig, Immunoglobulin.

### Peripheral Lymphocyte Subgroups and CD4^+^ T Cell Subsets

**Figure 1:**
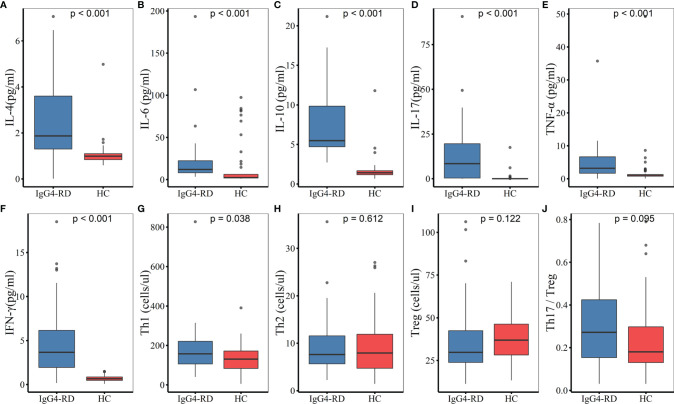
Comparisons of serum cytokines and CD4^+^ T cell subsets between IgG4-RD patients and HC. **(A**–**F)** The levels of serum IL-4, IL-6, IL-10, IL-17, TNF-α, and IFN-γ were higher in patients with IgG4-RD compared to HC (P < 0.001). **(G)** The absolute Th1 counts were higher in IgG4-RD patients than in HC (P < 0.05). **(H–J)** There was no significant difference in the absolute counts of Th2 and Treg cells, as well as Th17/Treg ratio between IgG4-RD patients and HC. HC, healthy controls.

Among the 52 IgG4-RD patients, 38 carried out the detection of peripheral lymphocyte subgroups, and 36 conducted CD4^+^ T cell subsets assay (**Table 5**). The absolute Th1 counts were higher in patients with IgG4-RD than in HC (157.58 cells/μl *vs.* 130.54 cells/μl, *P* = 0.038), whereas, there was no difference in percentages of Th1 cells between IgG4-RD patients and HC (20.01% *vs.* 16.9%, *P* = 0.207). The absolute counts of CD3^+^ T cells, CD19^+^ B cells, NK cells, CD4^+^ T cells, CD8^+^ T cells, Th2, Th17, and Treg cells showed no significant difference between patients with IgG4-RD and HC. However, the percentages of Treg cells was lower in IgG4-RD patients as compared with HC (3.64% *vs.* 5.19%, *P* = 0.002). No difference was observed in Th1/Th2 (20.02 *vs.* 15.7, *P* = 0.063) and Th17/Treg ratio (0.27 *vs.* 0.18, *P* = 0.095) between IgG4-RD patients and HC (**Figure 1**).

**Table 5:** Lymphocyte subgroups and CD4^+^ T cell subsets in patients with IgG4-RD.

Lymphocyte subsets	IgG4-RD	HC	*P-*value
CD3^+^ T (cells/μl)	1395.95 ± 540.92	1369.98 ± 340.74	0.800
CD19^+^ B (cells/μl)	158 (116, 218.75)	186.59 (138.76, 243.92)	0.266
NK (cells/μl)	251.78 (167.25, 303.5)	272.94 (172.24, 384.89)	0.353
CD4^+^ T (cells/μl)	824.77 (567.25, 1080.75)	716.22 (592.52, 848.91)	0.264
CD8^+^ T (cells/μl)	396 (308.25, 590.99)	464.24 (366.47, 694.87)	0.167
Th1 (cells/μl)	157.58 (105.7, 220.79)	130.54 (83.17, 171.6)	0.038
Th1 (%)^a^	20.01 (14.26, 24.20)	16.91 (12.25, 22.36)	0.207
Th2 (cells/μl)	7.61 (5.64, 11.55)	7.94 (4.7, 11.88)	0.612
Th2 (%)^b^	0.99 (0.77, 1.27)	1.01 (0.73, 1.47)	0.746
Th17 (cells/μl)	7.71 (4.32, 11.04)	7.1 (4.96, 9.25)	0.444
Th17 (%)^c^	1.00 (0.66, 1.42)	1.00 (0.61, 1.31)	0.986
Treg (cells/μl)	29.74 (23.84, 42.39)	36.88 (28.19, 46.26)	0.122
Treg (%)^d^	3.64 (3.17, 5.28)	5.19 (4.13, 6.33)	0.002
Th1/Th2	20.02 (14.66, 31.99)	15.7 (9.21, 22.43)	0.063
Th17/Treg	0.27 (0.15, 0.42)	0.18 (0.13, 0.3)	0.095

Data are expressed as mean ± S.D. or M (P_25_, P_75_). Lymphocyte subgroups (n=38) included CD3^+^ T, CD19^+^ B, NK, CD4^+^ T, CD8^+^ T; CD4^+^ T cell subsets (n=36) included Th1, Th2, Th17, Treg; HC, healthy controls (n=50); ^a^Percentages of Th1 in CD4^+^ T cells; ^b^Percentages of Th2 in CD4^+^ T cells; ^c^Percentages of Th17 in CD4^+^ T cells; ^d^Percentages of Treg in CD4^+^ T cells.

### Cytokines

Serum cytokines were measured in 25 of the 52 patients. Compared with HC, the levels of serum IL-4, IL-6, IL-10, IL-17, TNF-α, and IFN-γ were statistically higher in patients with IgG4-RD (P < 0.001). There was no significant difference in IL-2 levels between IgG4-RD patients and HC (**Figure 1**).

### Correlation Analysis

**Figure 2:**
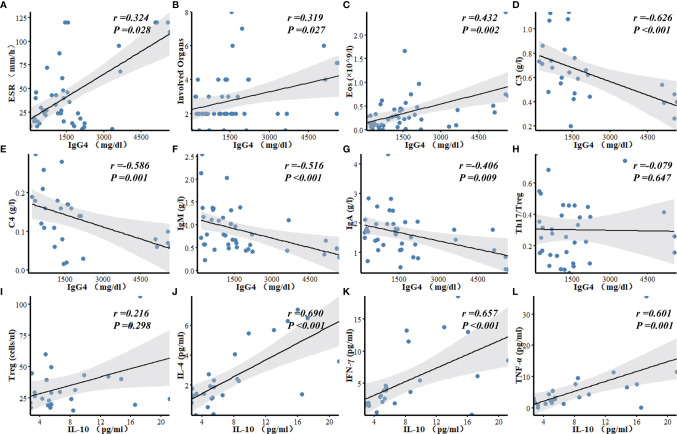
Correlation analyses of laboratory findings in patients with IgG4-RD. **(A**–**G)** Serum IgG4 level was positively correlated with ESR, the number of involved organs, and Eos, and negatively correlated with C3, C4, IgM, and IgA. **(H)** There was no correlation between serum IgG4 level and Th17/Treg ratio in IgG4-RD patients. **(I**–**L)** Serum IL-10 was uncorrelated with Treg, but positively correlated with IL-4, IFN-γ and TNF-α. Eos, eosinophils; C3, Complement 3; C4, Complement 4; Ig, Immunoglobulin.

**Figure 3:**
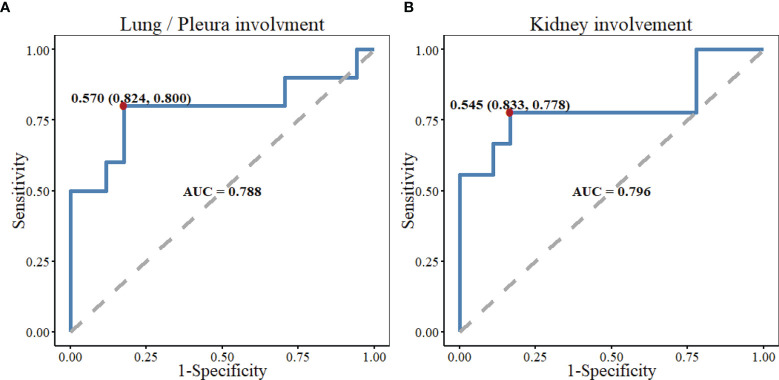
The predictive performance of serum C3 level in lung/pleura and kidney involvement in patients with IgG4-RD. **(A)** ROC test indicated the possibility of lung/pleura involvement when C3 was less than 0.570 g/l (AUC=0.788, P=0.014). **(B)** There might be kidney involvement when C3 was less than 0.545 g/l (AUC=0.796, P=0.014). C3, Complement 3; ROC, receiver operator characteristic; AUC, area under the curve; Kidney involvement included kidney, renal pelvis, and ureter involvement.

Serum IgG4 level was positively correlated with the number of involved organs (*r* = 0.319, *P* = 0.027), eosinophil counts (*r* = 0.432, *P* = 0.002), and ESR (*r* = 0.324, *P* = 0.028) in patients with IgG4-RD, and negatively correlated with the levels of serum C3 (*r* = -0.626, *P* < 0.001), C4 (*r* = -0.586, *P* = 0.001), IgM (*r* = -0.516, *P* < 0.001), and IgA (*r* = -0.406, *P* = 0.009). There was no correlation between serum IgG4 level and Th17/Treg ratio (**Figure 2**). Serum C3 level was a predictor of lung/pleura and kidney involvement in IgG4-RD patients. ROC test indicated the possibility of lung/pleura involvement when C3 was less than 0.570 g/l (*AUC* = 0.788, *P* = 0.014), and kidney involvement when C3 was less than 0.545 g/l (*AUC* = 0.796, *P* = 0.014) (**Figure 3**). Serum IL-10 was uncorrelated with Treg cells (*P* = 0.298), but positively correlated with IL-4, TNF-α, and IFN-γ (*P* ≤ 0.001) (**Figure 2**).

## Discussion

Our study described the demographic characteristics, laboratory examinations, and imaging features of 52 IgG4-RD patients, and focused on the analyses of T lymphocyte subsets and cytokine levels. Consistent with other reports (7, 8, 10, 11), the majority of patients in our cohort were middle aged to elderly Chinese men. Our findings confirmed that IgG4-RD often involved multiple organs successively or simultaneously, and ≥ 2 organs accounted for 80.8% of the cases. Submandibular gland, lacrimal gland/orbit, lung/pleura, pancreas, parotid, bile duct, and kidney were commonly involved organs, with less common ones including nasal cavity/sinuses, retroperitoneal fibrosis, skin, prostate, aorta, thyroid, hypophysis, meninges, mesentery, breast, and vagina.

Patients with elevated serum IgG4 levels accounted for 97.9% of the cases in our research, consistent with the findings of Yamada (7) and Lin (10). However, in the studies by Wallace (8) and Campochiaro (12), only 51% and 73% of the patients had elevated serum IgG4. This distinction may be related to the differences of region and ethnicity. Elevated serum IgG4 is a typical clinical manifestation of IgG4-RD, but elevated IgG4 only occurs in 51-98% of the patients, especially in Asian. Thus, it is imperative to integrate clinical, serological, radiological, and pathological features to make a comprehensive diagnosis of IgG4-RD.

IgG1, IgG2, and IgG3 increased to varying degrees in some patients, of which IgG2 increase accounted for 46.2% of the patients, significantly higher than that of IgG1 and IgG3. A retrospective study found that when IgG2 > 5.3g/l, the sensitivity of orbital IgG4-RD diagnosis can reach 80% and the specificity can reach 91.7%. The combined detection of serum IgG2 and IgG4 can be used as a new reference index for the diagnosis of IgG4-RD (13).

Our study found no significant difference in peripheral eosinophil counts between atopic and non-atopic patients. The absolute eosinophil counts were positively correlated with serum IgG4 level, consistent with the findings of Della-Torre (14). Regardless of whether the patient had an atopic disease, the histological specimens of the affected tissues tended to increase eosinophils. The evidence demonstrated that the elevated eosinophil counts seem not to be related to the presence of atopic diseases, but inherent to IgG4-RD itself (14–16).

Many similarities exist between IgG4-RD and allergic diseases, such as the increase of eosinophil counts, IgE, IgG4, and Th2 associated cytokines (IL-4, IL-5, and IL-13). However, no significant difference in serum IgG4 level, absolute Th2 counts and eosinophil counts between atopic and non-atopic patients was found in our patient cohort. Some scholars have also suggested that IgG4-RD and allergic disorders are not clinical manifestations of a single disease, but rather distinct disease processes that both involve type 2 immune activation. In Mattoo’s study (15, 17), only about 30% of IgG4-RD patients had an allergic history, while CD4^+^GATA3^+^ Th2 cells in the affected tissues were relatively sparse. Furthermore, another important study observed the expression of IL-4 mRNA increased in IgG4-RD tissues, and yet GATA3^+^ Th2 mRNA decreased, indicating that IL-4 was probably produced by non-Th2 cells (18). However, some studies showed the opposite conclusion. In IgG4-related Sclerosing Cholangitis and type 1 Autoimmune Pancreatitis (IgG4-SC/AIP) and Chronic Periaortitis (CP) patients, a dominant infiltration of GATA3^+^ Th2 cells were observed in the affected tissues (19, 20). With the further understanding of IgG4-RD, the role of Th2 cells in the pathogenesis will eventually be revealed.

The absolute Th1 counts were significantly increased in our study, which has also been reported in prior research (21). CD4^+^GZMA^+^IFN-γ^+^ CTLs were the most abundant infiltrating T lymphocytes in the IgG4-RD affected tissues, which could synthesize and secrete profibrotic cytokines of IL-1β, TGF-β1 and IFN-γ, as well as release cell-lysing substances such as perforin, granzyme A, and granzyme B (17, 18, 22). Elevated Th1 cells in our reaserch were probably part of CD4^+^GZMA^+^IFN-γ^+^ CTLs which have been misclassified as Th1 cells. CD4^+^ CTLs are not specific to IgG4-RD and also expand in the diseases of inflammatory bowel disease, systemic sclerosis, and rheumatoid arthritis, suggesting that these cells may play a role in the process of chronic inflammation and fibrotic disorder (9, 23, 24).

Treg cells play a vital role in maintaining immune homeostasis and autoimmune tolerance. Quantitative and functional defects of Treg cells have been described among a variety of autoimmune diseases (25–27). Intensive studies also have reported that the imbalance of Th17/Treg has a relationship with some autoimmune diseases (28–30). However, we found no difference in absolute Treg cells and Th17/Treg ratio between IgG4-RD patients and HC, consistent with Mattoo’s reports (22). In their study, 15 IgG4-RD patients received rituximab for 3-4 months, with dramatic clinical improvement, accompanied by a greater than 50% reduction in circulating CD4^+^ CTLs numbers and tissue fibrosis improved. Nonetheless, there was minimal impact on the number of circulating Treg cells. Based on the research above, it was suggested that Treg cells may not play a dominant role in the pathogenesis of IgG4-RD.

Cytokines are important small molecules in signal transmission between immune cells. As pro-inflammatory cytokines, the mild increases of serum IL-6, IL-17, TNF-α, and IFN-γ were in accordance with the mild inflammatory conditions in IgG4-RD. The slight increase of IL-4 may be related to the increase of Tfh2 (T follicular helper 2) cells (31, 32). IL-10, a representative anti-inflammatory cytokine, is mainly secreted by Treg cells and plays an important role in maintaining immune homeostasis. It was also mildly elevated and positively correlated with IL-4, TNF-α, and IFN-γ, but uncorrelated with Treg cells. It is assumed that the source of increased IL-10 does not exclude the participation of the other important cells such as Tfh cells (33). IL-10 may also play a pro-inflammatory role in IgG4-RD by promoting the survival and proliferation of B cells, production of antibodies, and immunoglobulin class switching.

The limitations of our study include the fact that it was a retrospective study with a relatively small sample size, and a few of the cases have a certain degree of data missing. In addition, due to the retrospective nature, we failed to investigate some other important data such as plasmablasts, Tfh cells, serum IgE, TGF-β, and disease activity assessed by IgG4-RD responder index (RI).

In conclusion, IgG4-RD is more common in middle aged and elderly men, and submandibular and lacrimal glands are the most frequently involved sites. Elevated Th1 cells probably play an important role in tissue lesion and fibrosis. Conversely, Th2 cells were unlikely to be linked to the pathogenesis of IgG4-RD. The quantitative defects of Treg cells and the imbalance of Th17/Treg may not be involved in the disease. Tfh cells, an important subset of CD4^+^ T cells, which play an essential role in germinal centre formation, B cells differentiation and IgG4 class-switching, hold a pivotal position in this disease process. That’s the focus of our further research. Following deeper understanding of IgG4-RD, we will eventually reveal the pathogenesis of this disease and improve the development of more precise and effective therapies.

## Data Availability Statement

The original contributions presented in the study are included in the article. Further inquiries can be directed to the corresponding author.

## Ethics Statement

The studies involving human participants were reviewed and approved by the Medical Ethics Committee of the second hospital of Shanxi Medical University (Shanxi, China). The patients/participants provided their written informed consent to participate in this study.

## Author Contributions

YY designed the study, analyzed the data, and wrote the manuscript. LS, SY and YL participated in the sample and data collection. CW provided Pathological specimen data and revised the manuscript. JL provided the healthy control data and revised the manuscript. CW designed the study and revised the manuscript. All authors contributed to the article and approved the submitted version.

## Funding

This work was supported by the National Natural Science Foundation of China (81971543, 81471618) and the Key Research and Development Projects of Shanxi Province, China (201803D31119).

## Conflict of Interest

The authors declare that the research was conducted in the absence of any commercial or financial relationships that could be construed as a potential conflict of interest.

## Publisher’s Note

All claims expressed in this article are solely those of the authors and do not necessarily represent those of their affiliated organizations, or those of the publisher, the editors and the reviewers. Any product that may be evaluated in this article, or claim that may be made by its manufacturer, is not guaranteed or endorsed by the publisher.
